# Patient Selection in Human Papillomavirus Related Oropharyngeal Cancer: The Added Value of Prognostic Models in the New TNM 8th Edition Era

**DOI:** 10.3389/fonc.2018.00273

**Published:** 2018-07-23

**Authors:** Sarah Deschuymer, Rüveyda Dok, Annouschka Laenen, Esther Hauben, Sandra Nuyts

**Affiliations:** ^1^Department of Radiation Oncology, KU Leuven-University of Leuven, University Hospitals Leuven, Leuven, Belgium; ^2^Laboratory of Experimental Radiotherapy, Department of Oncology, KU Leuven–University of Leuven, Leuven, Belgium; ^3^Leuven Biostatistics and Statistical Bioinformatics Center, KU Leuven-University of Leuven, Leuven, Belgium; ^4^Department of Imaging and Pathology, KU Leuven-University of Leuven, University Hospitals Leuven, Leuven, Belgium

**Keywords:** head and neck cancer, oropharyngeal cancer, human papillomavirus, TNM staging (8th edition), prognostic models, De-intensification trials

## Abstract

**Background:** With the growing interest in treatment de-intensification trials for human papillomavirus positive (HPV+) oropharyngeal squamous cell carcinoma (OPC), prognostic models have become essential for patient selection. The aim of this paper is to validate and compare the prognostic ability of the TNM 8th edition and previous published risk group classifications of Ang et al. and Rietbergen et al. and to derive a patient selection classification for de-intensification trials.

**Materials:** Patients with HPV+ OPC treated with curative (chemo)radiotherapy between 2004 and 2017 were classified according to the TNM 8th edition, the model of Ang et al. and of Rietbergen et al. HPV status was determined by p16 immunohistochemistry staining. Overall survival was estimated using the Kaplan-Meier method and groups were compared using the log-rank test. Harrell's C-index was used as measure of discriminative performance.

**Results:** A total of 333 OPC were identified of whom 100 were HPV+. The median follow-up was 63.7 months. The 5-year overall survival (5Y-OS) of stage I, II and III were 91.6, 55.2, and 38.0%. There was a significant difference between stage I vs. II and III. The Harrell's C-index for TNM 8th edition stage was 0.67. Including only HPV+ OPC, the Harrell's C-index for the model of Ang and Rietbergen were both 0.62. We combined the main prognostic factors defining the low risk groups in the three models, stage I, low comorbidity and ≤ 10 pack years, into one new low risk group to identify patients who may benefit from de-intensification trials. Intermediate risk was defined as stage I with high comorbidity or >10 pack years, high risk as stage II-III. The 5Y-OS were 100, 85.7, and 51.3%. The Harrell's C-index for the new classification model was 0.67.

**Conclusion:** Although TNM 8th edition provides better OS stratification than the 7th edition, it is not performant enough for patient selection, neither are the models from Ang et al. and Rietbergen et al. Therefore, we propose a patient selection classification for de-intensification trials based on the new TNM classification 8th edition, comorbidity and smoking pack years. In addition, this study emphasizes the importance of patient selection and personalized treatment for HPV+OPC.

## Introduction

Due to a change in etiology, oropharyngeal squamous cell carcinoma (OPC) is nowadays the shared name of two distinct clinical entities. First, OPC can be related to tobacco and alcohol consumption, which is also a well-known cause of multiple other types of cancer such as lung cancer or head and neck squamous cell carcinoma (HNSCC) in general. In the western world, the incidence of this type of OPC decreased by changing tobacco and alcohol habits. (1) Second, Human Papillomavirus (HPV) infection, transmitted by orogenital contact, can cause OPC. The incidence of HPV-driven OPC (also called HPV positive (HPV+) OPC) is on the rise, even to that extent that OPC is the only HNSCC subsite with a rising incidence and epidemiologic evidence has shown that it is getting epidemic proportions (2).

The prognosis of these two types of OPC is completely different. HPV+ OPC have a much better prognosis than tobacco and alcohol related OPC (further called HPV negative (HPV−) OPC). HPV+ OPC were often diagnosed as a stage III or IV [TNM 7th edition (7th Ed)] since they frequently present as a small primary tumor with multiple cervical lymph nodes. Such a stage IV (7th Ed) OPC had a 5-year overall survival (OS) around 70% in contrast to a 5-year OS of 30% for stage IV HPV− OPC (3).

Although these two types of OPC have different etiology and prognosis, the treatment for HPV+ and HPV− OPC is currently rather identical. Nevertheless, much ongoing research focuses on de-intensifying the treatment for HPV+ OPC aiming for equal tumor control and overall survival but with lower treatment related toxicity compared to the current standard treatment. With the increasing interest for these de-intensification trials, patient selection has become pivotal and the need for prognostic models and more accurate classification systems have emerged. In that perspective, the International Collaboration for Oropharyngeal Cancer Network on Staging (ICON-S) has developed new staging rules for better discrimination of stages for HPV+ OPC (4). Their proposed ICON-S classification was adopted in the new clinical (c) TNM 8th edition (8th Ed). The main shift lies in the grouping of the lymph nodes and of the stage groups. Consequently, more HPV+ OPC will be classified in stage I or II while stage IV is now reserved for metastasized OPC. It is important to keep in mind that this switch in staging not implies a switch in therapy, it currently only gives a better estimate of the outcome.

However, the outcome has not only been determined by TNM classification and HPV status but has additionally been influenced by patient related factors for example, smoking and comorbidities. In the last decade several different prognostic models have been developed and validated to take these influencing factors into account. These prognostic models have been either nomograms, calculating a specific percentage of probable survival, or risk group classifications. Ang et al. was one of the first reporting on a risk group classification with HPV status as independent prognostic factor for OS and progression-free survival. They classified patients with OPC in three risk groups of death based on four factors: HPV status, smoking pack years, T-stage and N-stage (7th Ed) (5). This classification of Ang et al. is generally the best known and has been validated by multiple authors (6–8). The main limitation of this classification was that all included patients were part of a randomized clinical trial (RTOG 1209). Consequently, this highly selected American patient population had a good performance status and were all advanced stage III and IV (7th Ed) OPC. In addition, it is known that the cigarette smoking habits in Europe differ from those in the United States (8). To overcome these limitations Rietbergen et al. developed and validated their prognostic risk model on unselected European OPC patients selecting HPV status, N-stage and comorbidity as risk group determinants (7, 8). The risk groups of Ang et al. and Rietbergen et al. were developed with the outdated T- and/or N-stage 7th Ed as influencing factors. The added value of these two prognostic models on the new TNM 8th Ed is currently unknown.

The aim of this project was first to validate the new cTNM 8th Ed in a Belgian HPV+ OPC population, second to investigate if there is still an added value of the previously published prognostic models from Ang et al. and Rietbergen et al. Third we combined these three different models in one new risk group classification to select patients for de-intensification trials.

## Materials and methods

### Patient and treatment characteristics

A retrospective chart review was performed of all non-metastatic oropharyngeal squamous cell carcinoma patients treated with primary radiotherapy (RT) in a single institution between January 2004 and July 2017. Patients with a synchronous second primary or with positive cervical lymph nodes without known primary tumor despite p16 positive status of the involved nodes were excluded from analysis. Local ethics committee approval was obtained before the start of the chart review.

Patients were scored for comorbidity, assessed with the adult comorbidity evaluation 27 (ACE27) (9), and for smoking pack years, categorized in never smokers, less than 10 pack years or more than 10 pack years. Alcohol consumption was not systematically reported in the patient charts and was therefore not further analyzed. The primary tumor volume of all HPV+ OPC was measured using the RT planning system (Varian Medical Systems, Palo Alto, CA).

All patients were treated with curative RT to an EQD2 of 70Gy. Locally advanced tumors (stage III and IV 7th Ed) were treated with concomitant chemoradiotherapy (CRT), cisplatin 100 mg/m^2^ 3-weekly, or RT concomitant with weekly cetuximab, an EGFR-inhibitor, if no contra-indications were present. Patients treated with primary surgery were excluded from analysis. Treatment was decided by the institutional multidisciplinary tumor board. After RT patients were followed every 2 months during the first 2 years, every 3 months in the third year, every 4 months in the fourth year, every 6 months in the fifth year and yearly thereafter.

### HPV testing

HPV status was evaluated by p16 immunohistochemistry (IHC) staining on formalin-fixed, paraffin-embedded tumor tissue. The biopsies were obtained during the diagnostic work-up of the patients. More than 70% diffuse nuclear and cytoplasmic staining was considered as HPV positive, less than 70% p16 staining was considered as HPV negative. P16 staining could not be performed if only fine needle aspiration cytology was obtained or if tissue blocks were not available. Patients with unknown HPV status were not eligible for analysis.

### TNM 7th and 8th edition

All HPV+ OPC were classified according to the TNM 7th Ed and to the cTNM 8th Ed. In the new cTNM 8th Ed, the T-stage remained practically unchanged apart from T4a and T4b (7th Ed.) combined to one T4-stage. N-stage, on the other hand, has changed extensively except for N0 and N3. Ipsilateral lymph nodes smaller than 6 cm independent of the number of suspicious lymph nodes, were all classified as N1. Bilateral or contralateral lymph nodes smaller than 6 cm were classified as N2 instead of N2c. Stage groups were stage I for T1T2 N0N1 tumors, stage II for T1-T3 N2 and T3 N0N1 tumors and stage III for T4 or N3 tumors. (Supplementary Material S1) Stage IV was reserved for metastatic disease independent of the primary T and N-classification.

HPV− OPC were only classified according to the TNM 7th Ed. In the new cTNM 8th Ed for HPV− OPC, a new criterion for N-stage is extranodal extension, defined as the presence of skin involvement or soft tissue invasion with deep fixation or tethering to underlying muscles or adjacent structures or clinical signs of nerve involvement. Lymph nodes with this extranodal extension are classified as cN3b, but this requires clinical evaluation of skin and soft tissue involvement which cannot be properly judged on a retrospective chart review. To avoid classification bias, we only validated the cTNM 8th Ed for HPV+ OPC.

### Risk group classification according to Ang and Rietbergen

**Figure 1:**
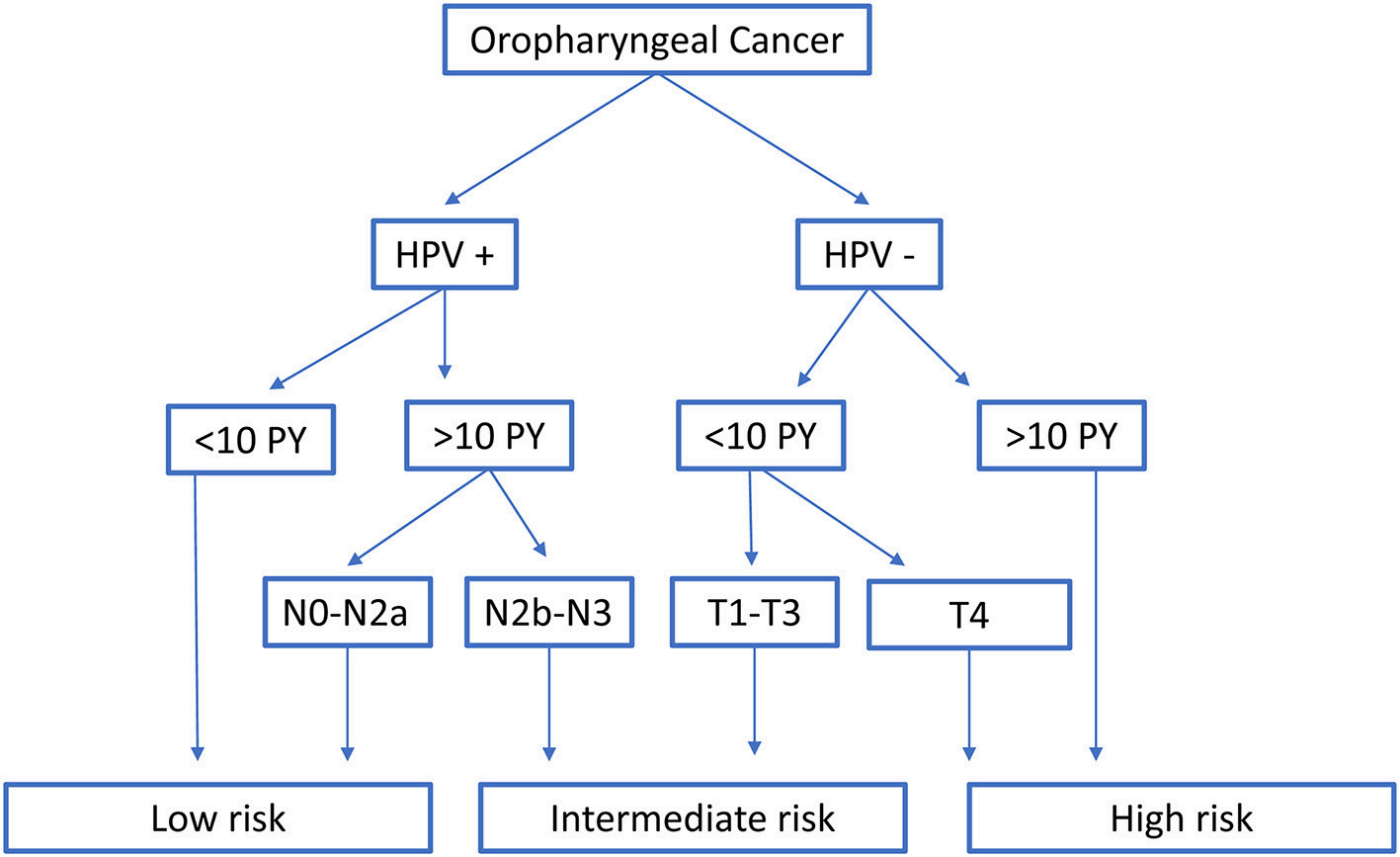
Classification into risk-of-death categories according to Ang et al. HPV, Human papillomavirus; PY, smoking pack years; T- and N-stage are according to the TNM 7th edition.

Both models from Ang et al. and Rietbergen et al. have classified OPC patients in three prognostic risk groups. Following the classification of Ang et al., all HPV + OPC with less than 10 smoking pack years or with N0-N2a-stage (7th Ed) were at low risk. Intermediate risk was reserved for HPV+ OPC with more than 10 smoking pack years and N2b-N3-stage (7th Ed), together with the HPV− OPC with less than 10 pack years and T1-T3-stage. HPV− OPC with more than 10 smoking pack years or with T4-stage were at high risk (5) (Figure 1).

**Figure 2:**
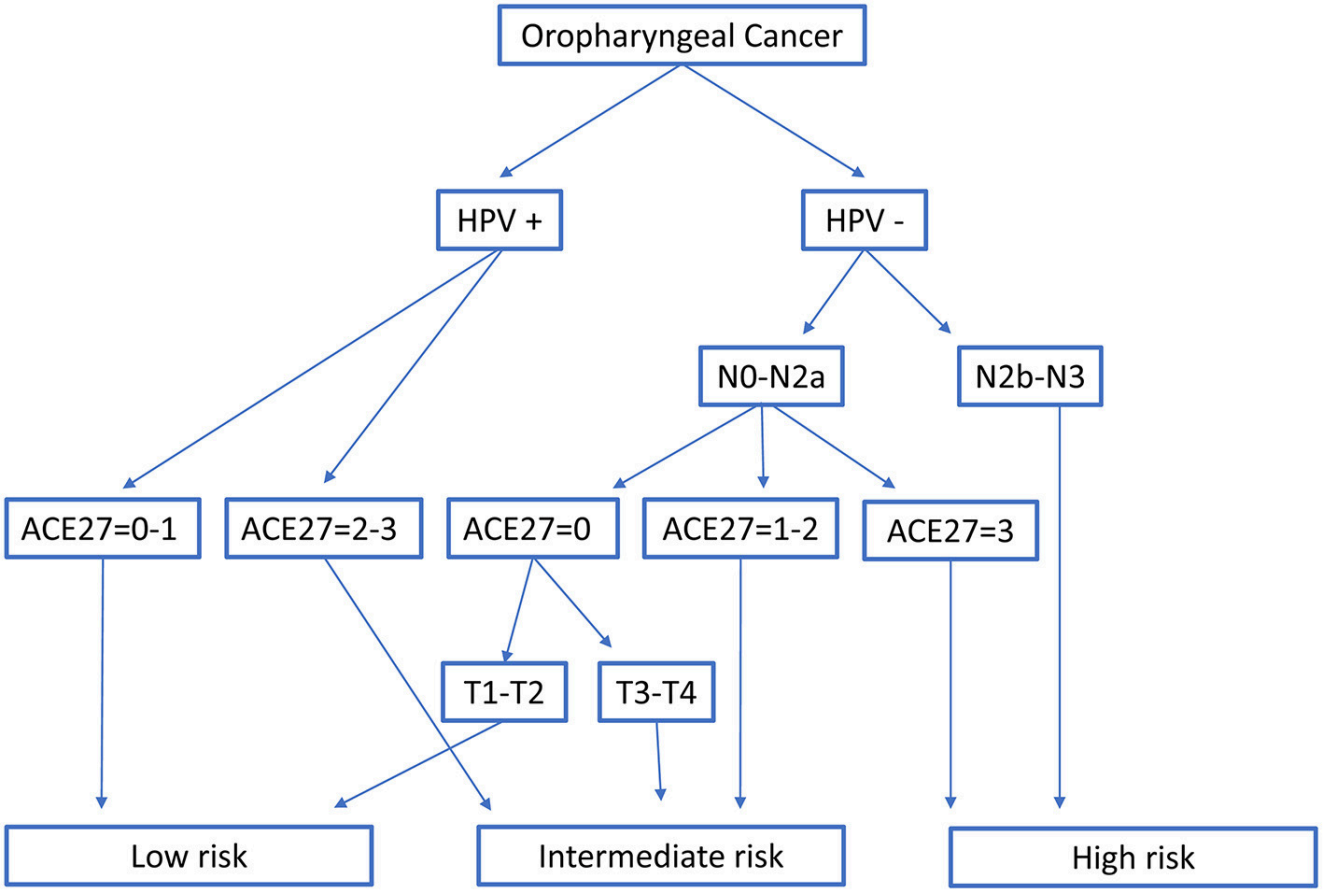
Classification into three risk-of-death categories according to Rietbergen et al. HPV, Human papillomavirus; T- and N-stage are according to the TNM 7th edition; ACE27, Adult Comorbidity Evaluation 27.

In Rietbergen et al. the HPV+ OPC were only divided by comorbidity index with ACE27 score of 0 or 1 considered as low risk and ACE27 score of 2 or 3 as intermediate risk. Classification in the low, intermediate or high risk group of HPV− OPC was based on N- stage (7th Ed), comorbidity index and T-stage (7th Ed) (8) (Figure 2).

### Statistics

Comparison of the patient and treatment characteristics of HPV− and HPV+ OPC was performed using the Fisher exact test for categorical variables and the Mann-Whitney U test for continuous variables. Overall survival (OS), locoregional control (LRC) and distant metastatic control (DMC) were calculated from the date of histological diagnosis to the date of death from any cause, to the date of locoregional relapse (tumor at the primary site or regional nodes) or to the date of distant metastases. OS rates were estimated by the Kaplan-Meier method and comparison between the curves was carried out using the log-rank test. The Cox regression model was used for analyzing the association between prognostic factors and OS. Given the limited number of events in the HPV+ OPC, a backward selection procedure was applied to construct a multivariable model. LRC and DMC were estimated by the cumulative incidence function using the Fine and Gray model, accounting for death as competing risk and were compared by Pepe and Mori test. The Harrell's concordance index (Harrell's C-index) was used as a measure of discriminative performance. The maximum value of the Harrell's C-index, 1, indicates a perfectly discriminating model while a value of 0.5 indicates that discrimination is not better than chance (10). All tests were two-sided, a 5% significance level was assumed for all tests. Analyses were performed using SAS software (version 9.4, SAS System for Windows).

## Results

### Patient characteristics

Patients charts of 333 patients with OPC were reviewed. P16 status could be determined of 260 patients of whom 100 (38.5%) were p16 positive and as a result considered HPV+. Patient characteristics of the 260 patients included in this study are listed in Table 1 separated according to HPV status. Smoking status and number of pack years could not be assessed in 2 HPV− OPC and 3 HPV+ OPC.

**Table 1:** Comparison patient and treatment characteristics of HPV negative and HPV positive patients.

	**HPV negative**	**HPV positive**	***P*-value**
**PATIENT AND TREATMENT CHARACTERISTICS**			
**Gender**			0.347
Male	130/160 (81.25%)	76/100 (76.00%)	
Female	30/160 (18.75%)	24/100 (24.00%)	
**Age**			**0.030**
Median (IQR)	59.7 (54.0-65.8)	63.6 (54.9-70.1)	
**Smoking**			**<0.001**
mean pack years (SD)	38.8 (22.12)	22.6 (21.65)	
Never smoker	3/160 (1.88%)	27/100 (27.00%)	
≤ 10 Pack years	8/160 (5.00%)	14/100 (14.00%)	
>10 Pack years	147/160 (91.87%)	56/100 (57.00%)	
Unknown	2/160 (1.25%)	3/100 (3.00%)	
**Systemic Treatment**			0.076
No	46/160 (28.75%)	31/100 (31.00%)	
Cisplatin	97/160 (60.63%)	66/100 (66.00%)	
EGFR-inhibitor	17/160 (17.00%)	3/100 (3.00%)	
**Comorbidity: ACE27**			**0.007**
0	26/160 (16.25%)	31/100 (31.00%)	
1	64/160 (40.00%)	37/100 (37.00%)	
2	38/160 (23.75%)	24/100 (24.00%)	
3	32/160 (20.00%)	8/100 (8.00%)	

*HPV, Human papillomavirus; IQR, Interquartile range; SD, Standard Deviation; ACE27, Adult Comorbidity Evaluation 27. Bold values p < 5% significance level*.

There were significantly more smokers and smokers with more than ten pack years in the HPV− group than in the HPV+ group. The mean number of pack years was also significantly higher in the HPV− group. Patients with HPV− OPC had significantly more comorbidity, measured with the ACE27 comorbidity index. The predominant TNM 7th Ed stage was stage IVa for HPV− as well as for HPV+ OPC. T-stage, N-stage and TNM group staging distribution (7th and 8th Ed) are shown in Table 2. The median follow-up time was 63.7 months (IQR 30.0; 99.9).

**Table 2:** Comparison between HPV negative and HPV positive patients according to the TNM 7th edition. Classification of the HPV positive patients according to the TNM 8th edition.

**T-stage (7th edition)**	**HPV negative**	**HPV positive**	***p*-value** 0.127	**T-stage (8th edition)**	**HPV positive**
T1	0/160 (0.00%)	12/100 (12.00%)		T1	12/100 (12.00%)
T2	53/160 (33.13%)	41/100 (41.00%)		T2	41/100 (41.00%)
T3	36/160 (22.50%)	19/100 (19.00%)		T3	19/100 (19.00%)
T4a	42/160 (26.25%)	23/100 (23.00%)		T4	28/100 (28.00%)
T4b	19/160 (11.88%)	5/100 (5.00%)			
**N-stage (7th edition)**			**0.018**	**N-stage (8th edition)**	
N0	37/160 (23.13%)	17/100 (17.00%)		N0	17/100 (17.00%)
N1	28/160 (17.50%)	10/100 (10.00%)		N1	57/100 (57.00%)
N2a	4/160 (2.50%)	5/100 (5.00%)		N2	20/100 (20.00%)
N2b	43/160 (26.88%)	42/100 (42.00%)		N3	6/100 (6.00%)
N2c	45/160 (28.13%)	20/100 (20.00%)			
N3	3/160 (1.88%)	6/100 (6.00%)			
**Stage (7th edition)**			0.504	**Stage (8th edition)**	
I	1/160 (0.63%)	0/100 (0.00%)		I	43/100 (43.00%)
II	19/160 (11.88%)	10/100 (10.00%)		II	23/100 (23.00%)
III	26/160 (16.25%)	11/100 (11.00%)		III	34/100 (34.00%)
IVa	92/160 (57.50%)	68/100 (68.00%)		IV	0/100 (0.00%)
IVb	22/160 (13.75%)	11/100 (11.00%)			

*HPV: Human papillomavirus. Bold font: p < 5% significance levels*.

### Survival

#### According to HPV status

**Figure 3:**
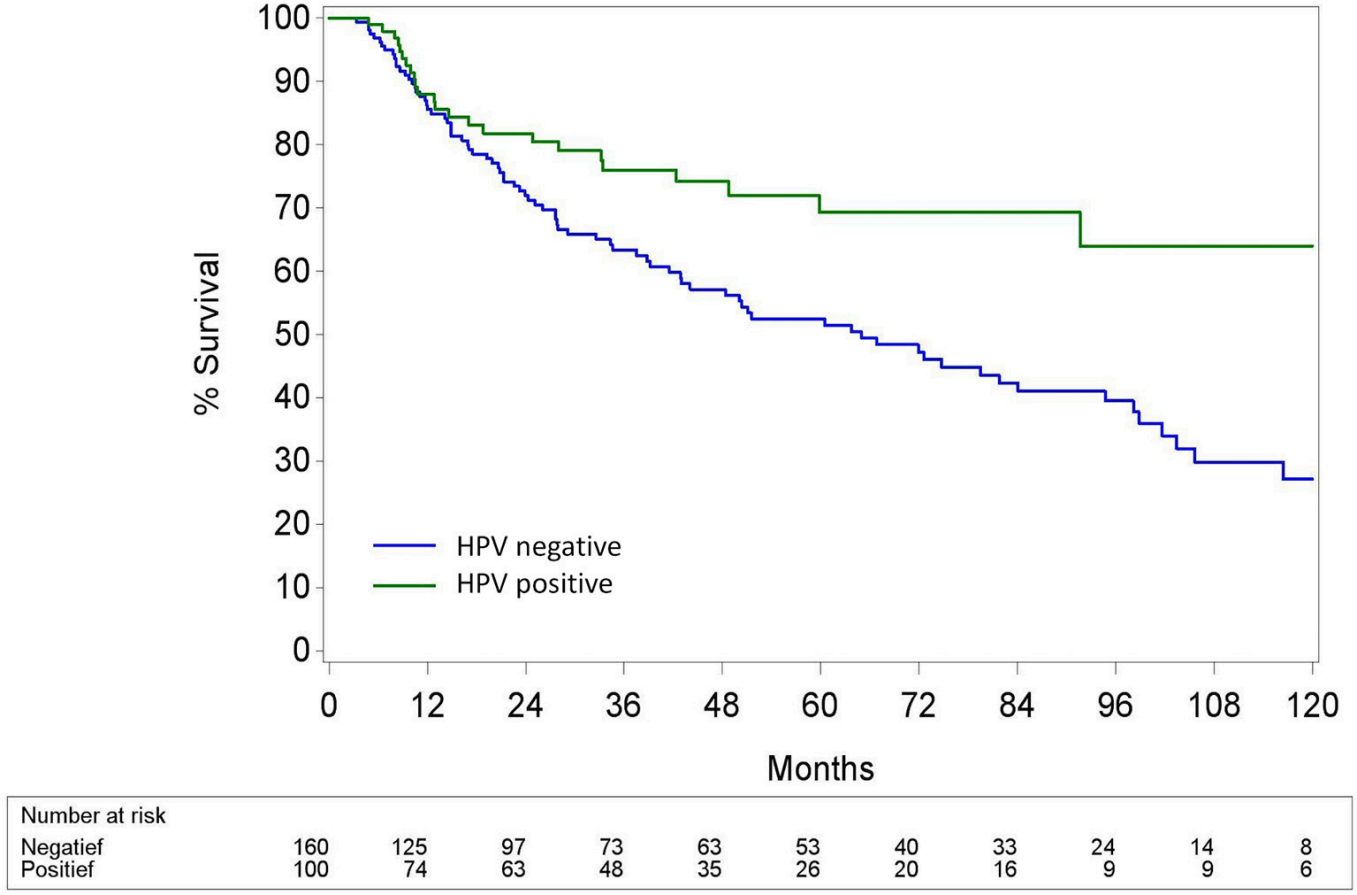
Kaplan-Meier curve for overall survival for HPV− and HPV+ Oropharyngeal squamous cell carcinoma. Log-rank test: *p* = 0.0018; HPV, Human papillomavirus.

**Figure 4:**
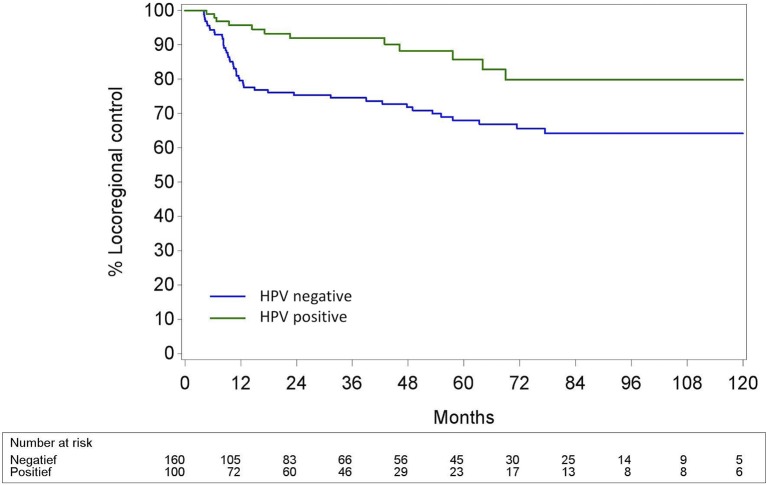
Locoregional control calculated with the cumulative incidence method with death as competing factor for HPV− and HPV+ Oropharyngeal squamous cell carcinoma. Pepe and Mori test: *p* = 0.0005; HPV, Human papillomavirus.

**Figure 5:**
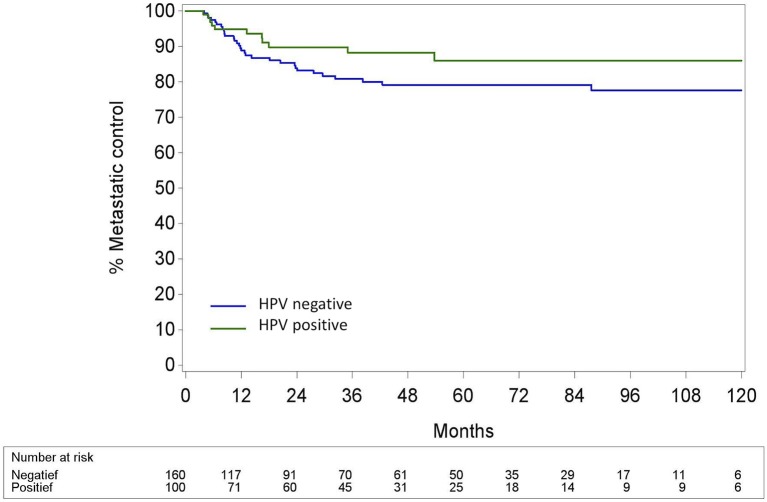
Distant metastatic control calculated with the cumulative incidence method with death as competing factor for HPV− and HPV+ Oropharyngeal squamous cell carcinoma. Pepe and Mori test: *p* = 0.14; HPV, Human papillomavirus.

The 5-year (5Y) overall survival (OS) was significantly better in the HPV+ OPC than in the HPV− OPC (69.3% (CI95% 56.6; 79.0) vs. 52.5% (CI95% 43.4; 60.7); *p* < 0.002). Locoregional control (LRC) was also significantly better in the HPV+ OPC with a 5Y-LRC of 85.8% (CI95% 76.0; 93.1) compared to the HPV− OPC [68.0% (CI95% 60.0.99; 75.7)] (*p* < 0.001). Distant metastatic control (DMC) of HPV+ and HPV− OPC was comparable [5Y-DMC 86.0% (CI95% 77.0; 92.8) and 79.1 % (CI95% 72.1; 85.4), respectively (*p* = 0.14)]. (Figures 3–5).

In univariable analysis, smoking pack years (*p* = 0.03), tumor volume (*p* = 0.003) and comorbidity (*p* = 0.009) were significant determinants of OS for HPV+ OPC but not for HPV− OPC. The use of concomitant cisplatin or cetuximab and age were not significant for neither HPV+ nor HPV− OPC. In multivariable analysis, tumor volume (*p* = 0.001) and comorbidity (*p* = 0.004) were independent predictors of OS for HPV+ OPC, while smoking pack years was borderline non-significant (Supplementary Material S2).

### According to the TNM 7th and 8th edition for HPV+ OPC

**Figure 6:**
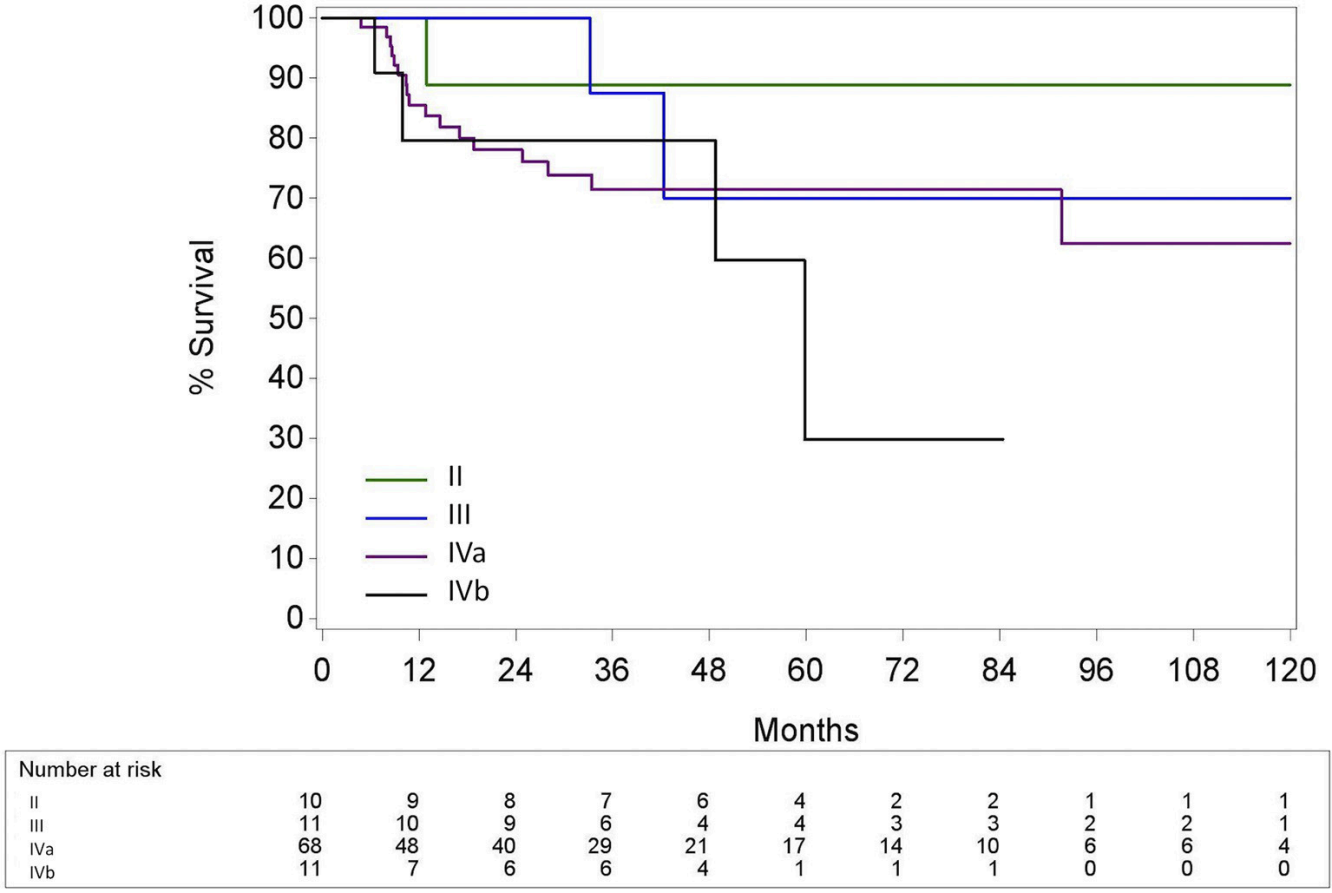
Kaplan-Meier curve for overall survival by stage group according to the TNM 7th edition for HPV positive oropharyngeal squamous cell carcinoma. Log-rank test: *p* = 0.39; HPV, Human papillomavirus.

**Figure 7:**
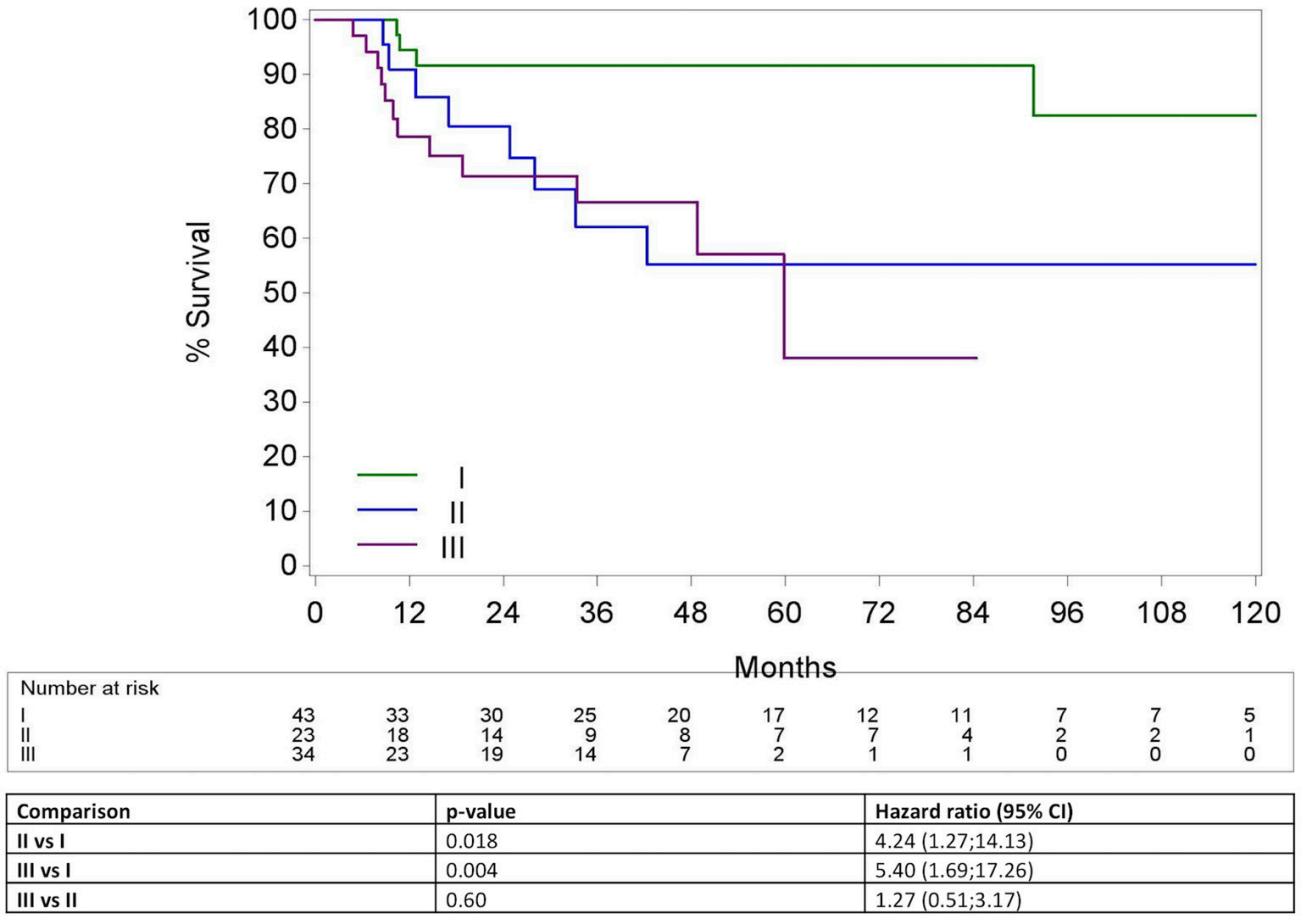
Kaplan-Meier curve for overall survival by stage group according to the TNM 8th edition for HPV positive oropharyngeal squamous cell carcinoma. Log-rank test: *p* = 0.006; Hazard Ratio >(<) 1 means higher (lower) risk for the first category; HPV, Human papillomavirus.

The 5Y-OS for TNM 7th Ed stage II, III, IVa and IVb were 88.9% (CI95% 43.3; 98.4); 70.0% (CI95% 22.5; 91.8); 71.4% (CI95% 57.3; 81.6) and 29.8% (CI95% 1.4; 71.1), respectively (*p* = 0.39) (Figure 6). In contrast to the TNM 7th Ed, there was a significant difference in OS between the different TNM 8th Ed stages with a 5Y-OS for TNM 8th Ed Stage I of 91.6% (CI95% 76.1; 97.2), stage II of 55.2% (CI95% 29.2; 75.1) and stage III of 38.0% (CI95% 8.7; 68.2) (*p* = 0.006) (Figure 7). On Cox regression analysis, OS was significantly lower for stage II compared to stage I [*p* = 0.018, Hazard ratio (HR) = 4.24 (CI95% 1.27; 14.13)] and for stage III compared to stage I [*p* = 0.004 HR = 5.40 (CI95% 1.69; 17.26)]. Nevertheless, there was no significant difference between stage II and III [*p* = 0.60, HR = 1.27 (CI95% 0.51; 3.17)]. The Harrell's C-index of the TNM 8th Ed was 0.67 (CI95% 0.57; 0.77).

### According to the risk groups of Ang et al. and Rietbergen et al.

**Figure 8:**
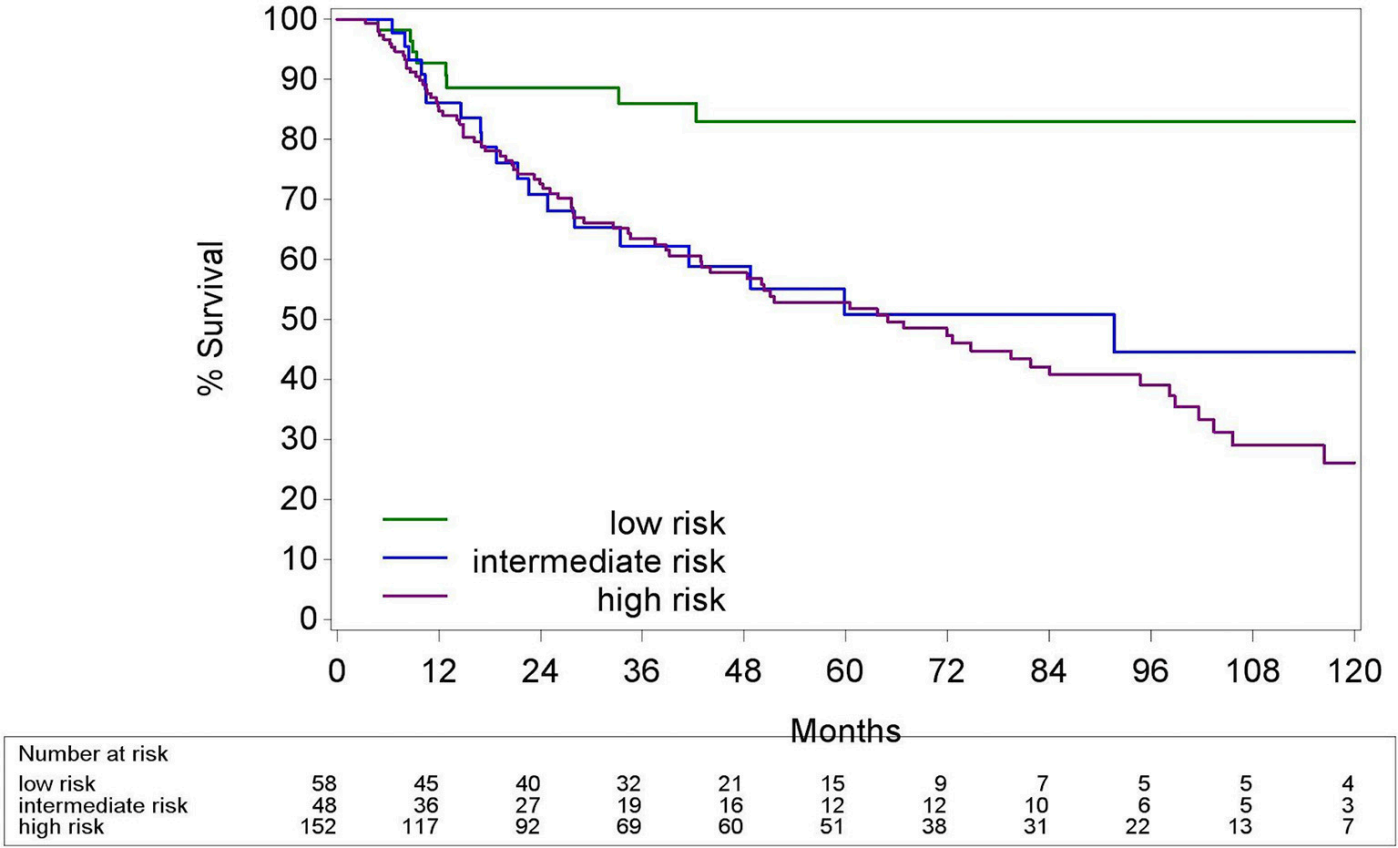
Kaplan-Meier curve of Overall survival of HPV− and HPV+ OPC by risk group according to Ang et al. HPV, Human papillomavirus.

**Figure 9:**
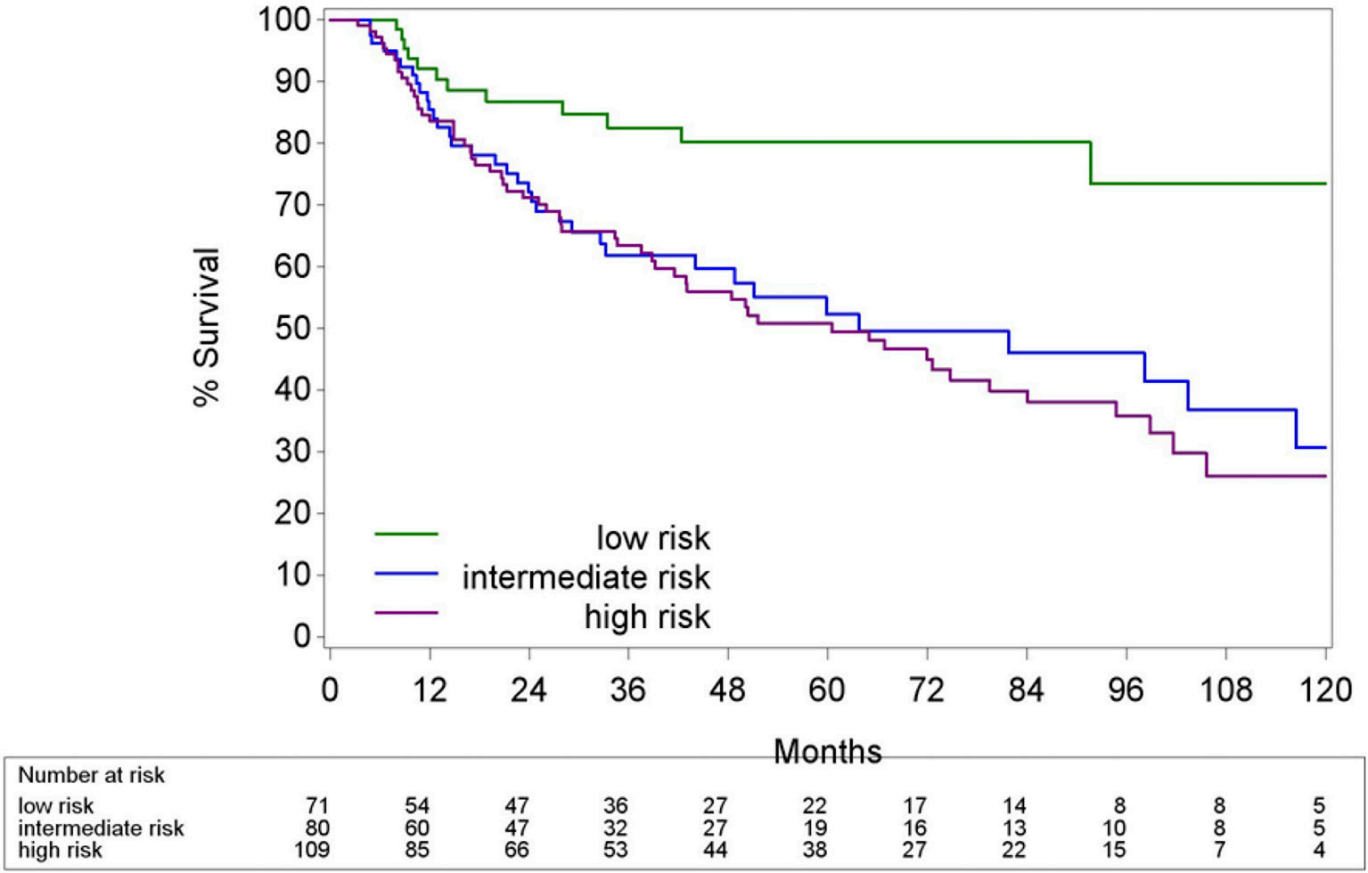
Kaplan-Meier curve of Overall survival of HPV− and HPV+ OPC by risk group according to Rietbergen et al. HPV, Human papillomavirus.

Classification of the patients into three risk groups as stated in Ang et al. and Rietbergen et al. is listed in Table 3. Two patients could not be classified according to the prognostic model from Ang et al. due to unknown number of pack years and N2b-N3 tumor (7th Ed). The 5Y-OS estimates for the low, intermediate and high risk group according to Ang et al. were 82.9, 50.9, and 52.8%, respectively. The Harrell's C-index was 0.57 (CI95%: 0.52; 0.62). The patients stratified according to Rietbergen et al. had a 5Y-OS of 80.2% in the low risk group, 52.3% in the intermediate risk group and 50.8% in the high risk group. Harrell's C-index was 0.58 (CI95% 0.53; 0.64). The differences between the low risk group compared with the intermediate and high risk group in both models were highly significant (Figures 8, 9 and Table 4).

**Table 3:** Classification of the included patient according to the risk groups of Ang et al. and of Rietbergen et al.

		**All patients**	**HPV negative**	**HPV positive**
Ang et al.	Low risk	58/258 (22.48%)		58/98 (59.18%)
	Intermediate risk	48/258 (18.60%)	8/160 (5.00%)	40/98 (40.82%)
	High risk	152/258 (58.91%)	152/160 (95.00%)	
Rietbergen et al.	Low risk	71/260 (27.31%)	3/160 (1.88%)	68/100 (68.00%)
	Intermediate risk	80/260 (30.77%)	48/160 (30.00%)	32/100 (32.00%)
	High risk	109/260 (41.92%)	109/160 (68.12%)	

*HPV, Human papillomavirus*.

**Table 4:** Cox regression analysis for overall survival by three risk groups according to Ang et al. or Rietbergen et al.

	**All OPC**		**Only HPV positive**	
	***P*-value**	**Hazard ratio (95% CI)**	***P*-value**	**Hazard ratio (95% CI)**
**Ang et al**.				
Intermediate vs. low risk	**0.007**	3.12 (1.36; 7.13)	**0.011**	3.07 (1.30; 7.26)
High vs. low risk	**<0.001**	3.75 (1.82; 7.78)		
High vs. intermediate risk	0.466	1.21 (0.73; 1.99)		
**Rietbergen et al**.				
Intermediate vs. low risk	**0.005**	2.50 (1.32; 4.73)	**0.009**	2.93 (1.30; 6.57)
High vs. low risk	**<0.001**	3.00 (1.64; 5.46)		
High vs. intermediate risk	0.392	1.20 (0.79; 1.83)		

*HPV+ patients are only classified in the low or intermediate risk group. Hazard ratio >(<1) means higher (lower) risk for the first category. CI, Confidence interval; OPC, oropharyngeal squamous cell carcinoma. Bold values p < 5% significance level*.

As the purpose was to compare these two models with the TNM 8th Ed for HPV+ OPC, we redid the analysis including only the HPV+ OPC. In both models HPV+ OPC were never classified in the high risk group. The 5Y-OS for the low risk group defined by Ang et al., HPV+ OPC with less than 10 pack years or N0-N2a-stage (7th Ed), was 82.9% (CI95% 68.2; 91.3) vs. 52.2% (CI95% 31.8; 69.2%) for the intermediate risk group, HPV+ OPC with more than 10 pack years and N2b-N3-stage, resulting in a Harrell's C-index of 0.62 (CI95% 0.51; 0.73). Applying the model of Rietbergen et al. the 5Y-OS for the low risk group, HPV+ OPC with ACE 27 score 0 or 1, was 81.1% (CI95% 67.3; 89.5) and for the intermediate risk group, HPV+ OPC with ACE 27 score 2 or 3, 44.9% (CI95% 21.4; 65.9), with a Harrell's C-index of 0.62 (CI95% 0.52; 0.73).

### According to a new risk group model

**Figure 10:**
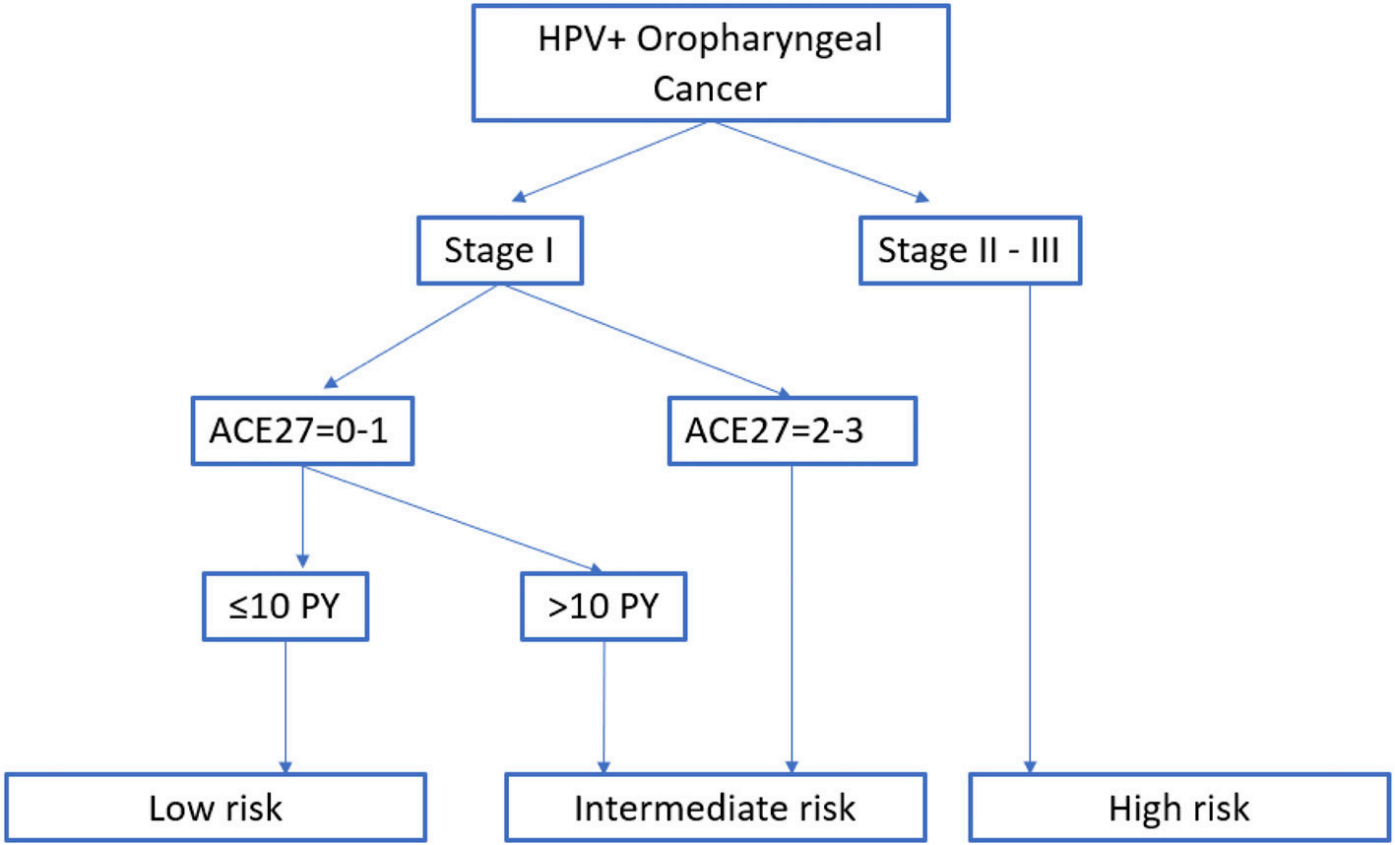
New proposed risk group model: classification into three risk-of-death categories. Stage groups I-III are according to the TNM 8th edition. HPV, Human papillomavirus; PY, smoking pack years; ACE27, Adult Comorbidity evaluation 27.

**Figure 11:**
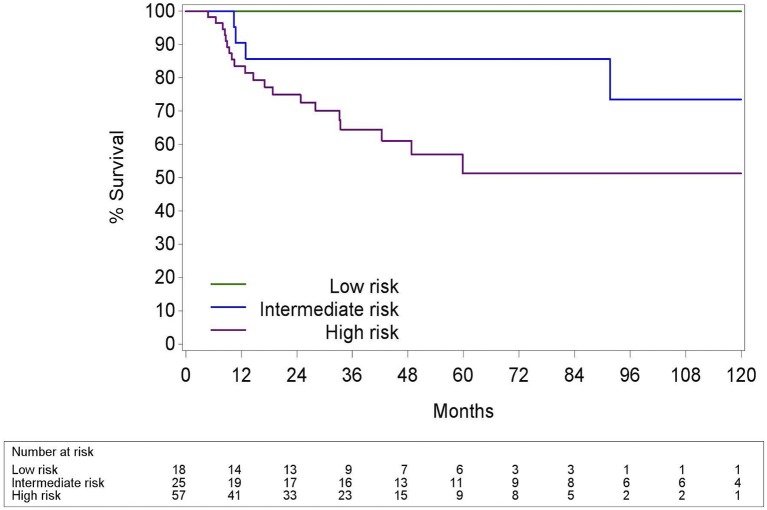
Kaplan-Meier curve for overall survival by risk groups defined in Figure 10 for HPV positive oropharyngeal squamous cell carcinoma. Log-rank test: *p* = 0.005; HPV, Human papillomavirus.

We propose a new risk group classification system based on the prognostic factors for HPV+ OPC in the three validated prognostic models: tumor stage 8th Ed, comorbidity (ACE27 0–1 vs. 2–3) and smoking (≤ of > than 10 pack years) (Figure 10). We combined the main prognostic factors defining the low risk groups in the three models, stage I, ACE 27 0-1 and less than 10 pack years, into one new low risk group to identify the patients who we believe may benefit from de-intensification trials. The intermediate risk group was defined as stage I with ACE27 score 2-3 or more than 10 smoking pack years and the high risk group as stage II or III. Patient and tumor characteristics of the different risk groups are listed in the Supplementary Material S3. The 5Y-OS following these risk groups was 100%, 85.7% (CI95% 62.0; 95.1) and 51.3% (CI95% 32.6; 67.2) (*p* = 0.005) (Figure 11). The Harrell's C-index was 0.67 (CI95% 0.60; 0.75). The 5Y-LRC following these risk groups was 100%, 82.3% (CI95% 60.7; 96.0) and 83.6% (70.05%; 93.4) (*p* = 0.004) (Supplementary Material S4).

Although the model of Ang et al. also stratifies according to N-stage (7th Ed), we did not incorporate this in our proposed risk groups as the N-stage division into N2a vs. N2b does not exist anymore in the new cTNM 8th Ed and we could not observe a significant difference either in OS between the different N-stages (Supplementary Data S5A). Moreover, the N-stage is indirectly incorporated in the TNM 8th Ed stage classification.

## Discussion

In this paper, we first investigated the prognostic impact of the new clinical TNM classification for HPV+ Oropharyngeal Cancer. The clinical TNM 8th Ed is indeed a better discriminator than the TNM 7th Ed for OS of HPV+ OPC following curative (C)RT and shows a clear survival difference between stage I vs. stage II and III. As expected, most of the HPV+ OPC were diagnosed as stage IV according to the TNM 7th Ed, while most HPV+ OPC were diagnosed as stage I according to the 8th Ed. Although, currently, this switch in stage classification does not yet lead to a shift in treatment, the psychological impact for the patients being diagnosed with a stage I instead of a stage IV disease and the opportunity to give accurate prognostic information are crucial for good patient care.

To our knowledge, this is the first European publication showing the improved prognostic and differentiating ability of the clinical TNM 8th Ed for patients treated exclusively with primary (C)RT. Although two of the seven centers in the original O'Sullivan et al. study were European, the ICON-S population mainly existed of a northern American population. As previous research has shown that the European and American population differ in terms of risk profile, it is valuable to validate the TNM in a European population (6, 8). Recently a German and Dutch research group explored and validated the prognostic ability of the TNM 8th Ed in 144 and 340 HPV+ OPC patients, respectively, however these patients were not exclusively treated with (C)RT (11, 12). The new TMN 8th Ed is divided into a clinical and a pathological staging system with differences in the N-stage and stage grouping. The clinical staging system focuses on size and laterality of the involved lymph nodes in contrast to the pathological (p) staging system focusing only on the number of involved lymph nodes (13). Patients can be staged in a different cN-stage than pN-stage and furthermore patients staged in the same cN-stage as pN-stage can be classified in different stage groups, for example cT3N2 and pT3N2 belong to stage II and stage III, respectively. Although in the original ICON-S paper of O'Sullivan 2% of the included patients were treated with surgery, we only included patients treated with curative primary RT, excluding stage IV (8th Ed), patients with metastatic disease at diagnosis, as they are not treated with curative intent.

Our results are in keeping with the findings of previous work by Australian researchers showing a significant OS difference between stage I and II and between I and III but not between stage II and III (14). Looking at our data, we could not find differences in patient nor treatment characteristics explaining the poor OS of stage II nor the lack of discriminating ability between stage II, including mainly T3 and N2 tumors, and III, including T4 and N3 tumors (Supplementary Material S1). We could not ascertain a significant difference in OS between N2 and N3-stage nor between T3 and T4-stage (Supplementary Material S5). Tumor volume, on the other hand, was a significant determinant for OS of HPV+ OPC in multivariable analysis. We hypothesize that tumor volume plays a more crucial prognostic role than T-stage. A previous published and validated nomogram of Velazquez et al. reported a better OS for T4 OPC compared to T3 OPC, supporting this hypothesis (10, 11). In our dataset, there was a significant difference in tumor volume between the different T-stages, nevertheless, there was no tumor volume difference between T3 and T4. This could explain the lack of OS difference between stage II and III (Supplementary Material S6). Tumor volume of larger datasets of HPV+ OPC should be analyzed in the future to establish the prognostic implications.

Albeit the prognostic ability of the 8th edition is better than the 7th edition, the TNM staging system alone is not performant enough for patient selection for de-intensifying studies. Previously, the risk group classification of Ang et al. and the European risk group classification of Rietbergen et al. have been proposed as patient selection models for de-intensification trials (7). We classified our HPV− and HPV+ OPC population according to these two validated prognostic models resulting in a rather poor Harrell's C-index of 0.58 and 0.57. To properly compare the models with the cTNM 8th Ed regarding the discrimination ability for HPV+ OPC, we redid the analysis excluding HPV− OPC. The Harrell's C-indices for both models improved, to the range of the discriminating power of the cTNM 8th Ed.

Overall, HPV+ OPC has a better OS and LRC than HPV− OPC. Nevertheless, a fraction of the HPV+ patients has a bad prognosis as seen in many publications, in daily clinical practice and in our data, more specifically, HPV+ OPC stage II and III, heavy smokers and patients with more comorbidity. In both models from Ang et al. and Rietbergen et al. there was no difference in OS between the intermediate and the high risk group, meaning that HPV+ OPC classified as intermediate risk had an equally poor prognosis as HPV− OPC. Including these HPV+ OPC patients with bad prognosis in de-intensification trials, could have detrimental consequences. Alternatively, these HPV+ OPC could benefit from intensification of the treatment for example, inclusion in RT dose escalation trials, instead of de-intensification trials. In this regard we like to point out the importance of patient selection and individualized treatment decisions to avoid undermining the overall survival of HPV+ patients by de-intensifying the treatment. Future research should focus on the development of more accurate prognostic models for HPV+ OPC as a unique disease including also biomarkers, genetic information and functional imaging.

Based on the prognostic determinants of the three afore-mentioned models we made a new risk group classification system. We propose the low risk group, patients with stage I HPV+ OPC with comorbidity ACE 27 0–1 and less than 10 pack years, as candidate patients to include in de-intensification trials. As the aim of this project was to investigate the added value of the prognostic models in addition to the cTNM 8th Ed, the cTNM 8th Ed was kept as the base of the classification model dividing the HPV+ OPC in two groups, stage I vs. II-III. Next, we separated stage I by comorbidity score since comborbidity was an independent predictor of overall survival in multivariable analysis and it defined the low risk group by Rietbergen et al. Last, the low risk group was limited to ≤ 10 pack years following the classification of Ang et al. As previously described, we did not separate our risk groups on N-stage like in the model of Ang et al. since the cTNM 8th Ed categorizes the previous N2a and N2b-stage (7th Ed) all as N1-stage. Interestingly, not all patients within the N1-stage (8th Ed) can be treated in the same manner as O'Sullivan et al. showed that HPV+ OPC with more than 10 pack years and N2b-stage (7th Ed) have a reduced distant control when treated with RT alone compared to CRT (15). Including these patients in de-intensification trials omitting chemotherapy should therefore be avoided. In consequence, we did not include patients with more than 10 pack years in our low risk group even though smoking pack years was only significant in univariable analysis. Smoking is believed to influence the tumor biology and to induce more hypoxia leading to diminished radio-sensitivity of the tumor (16). We observed a higher locoregional recurrence rate in the intermediate than the low risk group. However, because there were only 3 recurrences in the intermediate group, no statically significant difference between the intermediate and the low risk group could be established. In practice, several (but not all) running de-intensification trials include the number of maximum smoking pack years in their inclusion criteria. Nevertheless, it is currently unclear what the best cut-off of smoking pack years is and the exact influence of smoking pack years on the OS and LRC for HPV+ OPC is still under investigation.

Unfortunately, the Harrell's C-index of the new model was not better than the cTNM 8th Ed and our patient population was too small to make firm conclusions. Therefore, our proposed risk group classification is purely hypothesis generating and must be validated in the future. In spite of this, it points out the importance of patient selection in a HPV+ OPC population.

The strength of this paper is that all patients were treated with curative primary RT and all HPV+ OPC were classified according to the clinical TNM 8th Ed. Our study has a couple of limitations, particularly the retrospective nature of the data collection and the rather small fraction of HPV+ OPC influencing the strength of our analysis. The cTNM 8th Ed was additionally only validated in the HPV+ OPC and not for the HPV− OPC. Although, in practice, the TNM 8th Ed for HPV− OPC with the addition of N-stage N3b, will only change the group staging of a very restricted subgroup with a switch from stage III or IVa to IVb and will consequently only have minimal influence on the predicted prognosis.

We were unable to determine the p16 status of one fifth of the OPC patients. We are aware that some researchers prefer the combination of p16 IHC with HPV DNA PCR or *in situ* hybridization (ISH) to reduce the number of false positives, nevertheless, the best method for HPV detection and their prognostic implications remain controversial. Lewis et al. found that all p16 positive OPC, whether HPV positive by ISH, HPV negative by ISH alone, or HPV negative by ISH and PCR, showed statistically significant better overall and disease free survival than p16 negative OPC (17). In contrast, others demonstrated that p16 positive/ HPV PCR negative OPC have worse overall survival compared with p16 positive/HPV PCR positive OPC (8, 11). Notably, the number of patients in these subgroups with discrepancy in p16 IHC and ISH or PCR are all very small making firm conclusions difficult. We chose to classify the tumors as HPV+ by means of p16 immunohistochemistry for several reasons. First, this single detection method by p16 IHC is an easy and cost-effective surrogate marker for HPV infection with a high sensitivity and it was allowed for HPV identification in the original ICON-S population (4). Second, the Union for International Cancer Control (UICC) divides OPC in p16 positive or p16 negative and classifies them in their corresponding TNM classification, independent of their HPV status (18). Last, p16 IHC is standard of care in daily clinical practice in Belgium (19). Interestingly, there is no consensus HPV detection method in the running de-intensification trials.

## Conclusion

In summary, we report the validation of the cTNM 8th Ed for HPV+ OPC in our single center Belgian population treated with primary (chemo)radiotherapy. The prognosis of HPV+ OPC is additionally influenced by patient specific characteristics such as smoking pack years and comorbidity, demonstrated by the risk group classifications of Ang et al. and Rietbergen et al. We propose a new risk group classification combining the cTNM 8th Ed with these risk factors. The low risk group patients defined as stage I, never smokers or smokers with less than 10 pack years and low comorbidity have an excellent prognosis and may benefit from de-intensification trials. In addition, this study emphasizes the importance of patient selection and personalized treatment for HPV+ OPC.

## Ethics statement

This study was carried out in accordance with the recommendations of the ethics committee of the University Hospitals of Leuven. This protocol was approved by the ethics committee of the University Hospitals of Leuven. This manuscript involves research with human subjects and was conducted in accordance with the World Medical Association's Declaration of Helsinki. This retrospective study was exempt from patient written informed consent by the ethics committee of the University Hospitals of Leuven.

## Author contributions

Each author has provided substantial contributions to warrant authorship. SD and SN contributed conception and design of the study; SD organized the database; RD and EH performed the immunohistochemistry staining; AL and SD performed the statistical analysis; SD and SN wrote the manuscript. All authors contributed to manuscript revision, read and approved the submitted version.

### Conflict of interest statement

The authors declare that the research was conducted in the absence of any commercial or financial relationships that could be construed as a potential conflict of interest.
