# Supplementary material for: Patient Selection in Human Papillomavirus Related Oropharyngeal Cancer: The Added Value of Prognostic Models in the New TNM 8th Edition Era
Source: Front Oncol. 2018 Jul 23;8:273. doi: 10.3389/fonc.2018.00273 (PMC6065203; doi:10.3389/fonc.2018.00273)
Supplement: Supplementary file 1 [file Data_Sheet_1.PDF]

Supplementary Material

Title:

Patient Selection in Human Papillomavirus Related Oropharyngeal Cancer: The Added Value of Prognostic Models in the New TNM 8th Edition Era

Running title: Patient-selection in HPV+ Oropharyngeal Cancer

Sarah Deschuymer, Rüveyda Dok, Annouschka Laenen, Esther Hauben, Sandra Nuyts*.

*** Correspondence:** Sandra.nuyts@uzleuven.be

Supplementary Tables and Figures:

S1: Comparison of TNM 7th edition and 8th edition of the T- and N-stages and the number of included patients for each T- and N-stage.
S2: Predictors for overall survival in univariable **(A)** and multivariable **(B)** cox regression analysis.
S3: Patient and tumor characteristics separated by risk group according to the new proposed classification model.
S4: Locoregional control calculated with the cumulative incidence method with death as competing factor for the risk groups defined in figure 10.
S5: Kaplan-Meier curve for overall survival by N-stage **(A)** and T-stage **(B)** according to the TNM 8th edition for HPV positive oropharyngeal squamous cell carcinoma.
S6: Tumor volume of HPV+ OPC according to T-Stage.

S1: Comparison of TNM 7th edition and 8th edition of the T- and N-stages. T-stage remained practically unchanged apart from T4a and T4b (7th Ed.) combined to one T4-stage. N-stage, on the other hand, has changed extensively except for N0 and N3. Ipsilateral lymph nodes smaller than 6 cm independent of the number of suspicious lymph nodes, were all classified as N1. Bilateral or contralateral lymph nodes smaller than 6 cm were classified as N2 instead of N2c. Stage groups were stage I (green) for T1T2 N0N1 tumors, stage II (blue) for T1-T3 N2 and T3 N0N1 tumors and stage III (red) for T4 or N3 tumors. Stage IV was reserved for metastatic disease independent of the primary T and N-classification. The number of the included patients for each T- and N-stage are listed in the corresponding column or row.

**8th edition**		T1	T2	T3	**T4**	
	7th edition	T1	T2	T3	T4a	T4b
N0	N0	0	10	1	6	0
**N1**	N1	3	3	4	0	0
	N2a	0	5	0	0	0
	N2b	8	14	10	8	2
**N2**	N2c	1	5	2	9	3
N3	N3	0	4	2	0	0
