# Supplementary material for: Patient Selection in Human Papillomavirus Related Oropharyngeal Cancer: The Added Value of Prognostic Models in the New TNM 8th Edition Era
Source: Front Oncol. 2018 Jul 23;8:273. doi: 10.3389/fonc.2018.00273 (PMC6065203; doi:10.3389/fonc.2018.00273)
Supplement: Supplementary file 2 [file Data_Sheet_2.PDF]

Supplementary Material

Title:

Patient Selection in Human Papillomavirus Related Oropharyngeal Cancer: The Added Value of Prognostic Models in the New TNM 8th Edition Era

Running title: Patient-selection in HPV+ Oropharyngeal Cancer

Sarah Deschuymer, Rüveyda Dok, Annouschka Laenen, Esther Hauben, Sandra Nuyts*.

*** Correspondence:** Sandra.nuyts@uzleuven.be

Supplementary Tables and Figures:

S1: Comparison of TNM 7th edition and 8th edition of the T- and N-stages and the number of included patients for each T- and N-stage.
S2: Predictors for overall survival in univariable **(A)** and multivariable **(B)** cox regression analysis.
S3: Patient and tumor characteristics separated by risk group according to the new proposed classification model.
S4: Locoregional control calculated with the cumulative incidence method with death as competing factor for the risk groups defined in figure 10.
S5: Kaplan-Meier curve for overall survival by N-stage **(A)** and T-stage **(B)** according to the TNM 8th edition for HPV positive oropharyngeal squamous cell carcinoma.
S6: Tumor volume of HPV+ OPC according to T-Stage.

S2A: Predictors for overall survival in univariable cox regression analysis. Smoking was classified as never smoker, ≤10 pack years and >10 pack years. Systemic treatment was classified as no systemic treatment, cisplatin or EGFR-inhibitor. The p-value refers to global test. Tumor volume was only measured for the HPV positive Oropharyngeal Cancers; Hazard ratio >(<)1 means higher (lower) risk for the higher level. CI: confidence interval; ACE 27: Adult comorbidity evaluation 27; HPV: Human papillomavirus.

	**HPV negative**		**HPV positive**	
	**p-value**	**Hazard ratio (95% CI)**	**p-value**	**Hazard ratio (95% CI)**
**Smoking**	0.325	1.45 (0.69;3.04)	**0.032**	1.96 (1.06;3.63)
**Age**	0.323	1.01 (1.99;1.04)	0.081	1.04 (0.99;1.07)
**Systemic treatment**	0.103		0.578	
**ACE 27 (2/3 vs 0/1)**	0.199	1.32 (0.86;2.03)	**0.009**	2.93 (1.30;6.57)
**Tumor volume (+1cc)**	/	/	**0.003**	1.03 (1.01;1.05)

S2B: Predictors for overall survival in multivariable cox model. Given the limited number of events in the HPV+ OPC population, a backward selection procedure was applied to construct a multivariable model. Smoking pack years was borderline non-significant and hence removed from the model.

**Variable**	**p-value**	**Hazard ratio (95% CI)**
**Tumor Volume**	0.001	1.03 (1.01;1.05)
**ACE 27 (2/3 vs 0/1)**	0.004	3.48 (1.50;8.04)
