# Supplementary material for: Patient Selection in Human Papillomavirus Related Oropharyngeal Cancer: The Added Value of Prognostic Models in the New TNM 8th Edition Era
Source: Front Oncol. 2018 Jul 23;8:273. doi: 10.3389/fonc.2018.00273 (PMC6065203; doi:10.3389/fonc.2018.00273)
Supplement: Supplementary file 3 [file Data_Sheet_3.PDF]

frontiers

Supplementary Material

Title:

Patient Selection in Human Papillomavirus Related Oropharyngeal Cancer: The Added Value of Prognostic Models in the New TNM 8th Edition Era

Running title: Patient-selection in HPV+ Oropharyngeal Cancer

Sarah Deschuymer, Rüveyda Dok, Annouschka Laenen, Esther Hauben, Sandra Nuyts*.

*** Correspondence:** Sandra.nuyts@uzleuven.be

Supplementary Tables and Figures:

S1: Comparison of TNM 7th edition and 8th edition of the T- and N-stages and the number of included patients for each T- and N-stage.
S2: Predictors for overall survival in univariable **(A)** and multivariable **(B)** cox regression analysis.
S3: Patient and tumor characteristics separated by risk group according to the new proposed classification model.
S4: Locoregional control calculated with the cumulative incidence method with death as competing factor for the risk groups defined in figure 10.
S5: Kaplan-Meier curve for overall survival by N-stage **(A)** and T-stage **(B)** according to the TNM 8th edition for HPV positive oropharyngeal squamous cell carcinoma.
S6: Tumor volume of HPV+ OPC according to T-Stage.

S3: Patient and tumor characteristics by risk group according to the new proposed classification model. IQR: Interquartile range; ACE27: Adult Comorbidity Evaluation 27.

	**Low risk**	**Intermediate risk**	**High risk**
**Gender**			
Male	11/18 (61.11%)	20/25 (80.00%)	45/57 (78.95%)
Female	7/18 (38.89%)	5/25 (20.00%)	12/57 (21.05%)
**Age**			
Median (IQR)	63.4 (52.0-69.0)	61.5 (54.2-72)	63.5 (54.7-69.9)
**Smoking**			
Never smoker	11/18 (61.11%)	1/25 (4.00%)	15/57 (26.32%)
≤ 10 Pack years	7/18 (38.89%)	2/25 (8.00%)	5/57 (8.77%)
> 10 Pack years	-	20/25 (80.00%)	36/57 (63.16%)
Unknown		2/25 (8.00%)	1/57 (1.75%)
**Systemic Treatment**			
No	8/18 (44.44%)	8/25 (32.00%)	15/57 (26.32%)
Yes	10/18 (55.56%)	17/25 (68.00%)	42/57 (73.68%)
**Comorbidity: ACE27**			
0-1	18/18 (100%)	14/25 (56.00%)	36/57 (63.16%)
2-3	-	11/25 (44.00%)	21/57 (36.84%)
**Stage (7th edition)**			
I	-	-	-
II	5/18 (27.78%)	5/25 (20.00%)	-
III	4/18 (22.22%)	2/25 (8.00%)	5/57 (8.77%)
IV	9/18 (50.00%)	18/25 (72.00%)	52/57 (91.23%)
**Stage (8th edition)**			
I	18/18 (100%)	25/25 (100%)	-
II	-	-	23/57 (40.39%)
III	-	-	34/57 (59.62%)
