# Supplementary material for: Patient Selection in Human Papillomavirus Related Oropharyngeal Cancer: The Added Value of Prognostic Models in the New TNM 8th Edition Era
Source: Front Oncol. 2018 Jul 23;8:273. doi: 10.3389/fonc.2018.00273 (PMC6065203; doi:10.3389/fonc.2018.00273)
Supplement: Supplementary file 5 [file Data_Sheet_5.PDF]

Supplementary Material

Title:

Patient Selection in Human Papillomavirus Related Oropharyngeal Cancer: The Added Value of Prognostic Models in the New TNM 8th Edition Era

Running title: Patient-selection in HPV+ Oropharyngeal Cancer

Sarah Deschuymer, Rüveyda Dok, Annouschka Laenen, Esther Hauben, Sandra Nuyts*.

*** Correspondence:** Sandra.nuyts@uzleuven.be

Supplementary Tables and Figures:

S1: Comparison of TNM 7th edition and 8th edition of the T- and N-stages and the number of included patients for each T- and N-stage.
S2: Predictors for overall survival in univariable **(A)** and multivariable **(B)** cox regression analysis.
S3: Patient and tumor characteristics separated by risk group according to the new proposed classification model.
S4: Locoregional control calculated with the cumulative incidence method with death as competing factor for the risk groups defined in figure 10.
S5: Kaplan-Meier curve for overall survival by N-stage **(A)** and T-stage **(B)** according to the TNM 8th edition for HPV positive oropharyngeal squamous cell carcinoma.
S6: Tumor volume of HPV+ OPC according to T-Stage.

S5A: Kaplan-Meier curve for overall survival by N-stage according to the cTNM 8th edition for HPV positive oropharyngeal squamous cell carcinoma. Log-rank test: p=0.057; HPV: Human papillomavirus.

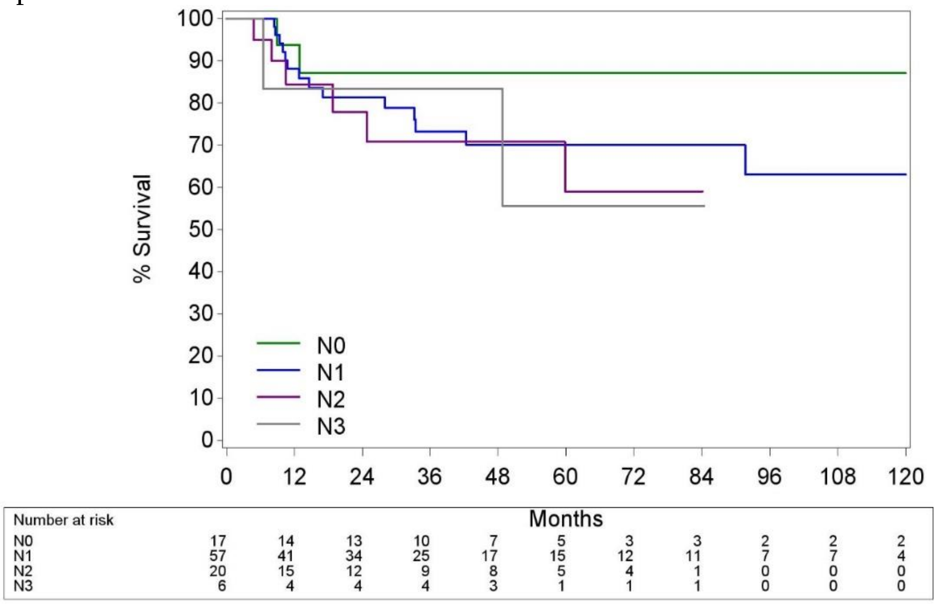

S5B: Kaplan-Meier curve for overall survival by T-stage according to the TNM 8th edition for HPV positive oropharyngeal squamous cell carcinoma. Global log-rank test: p=0.019. Hazard Ratio >(<) 1 means higher (lower) risk for the first category. CI: confidence interval; HPV: Human papillomavirus.

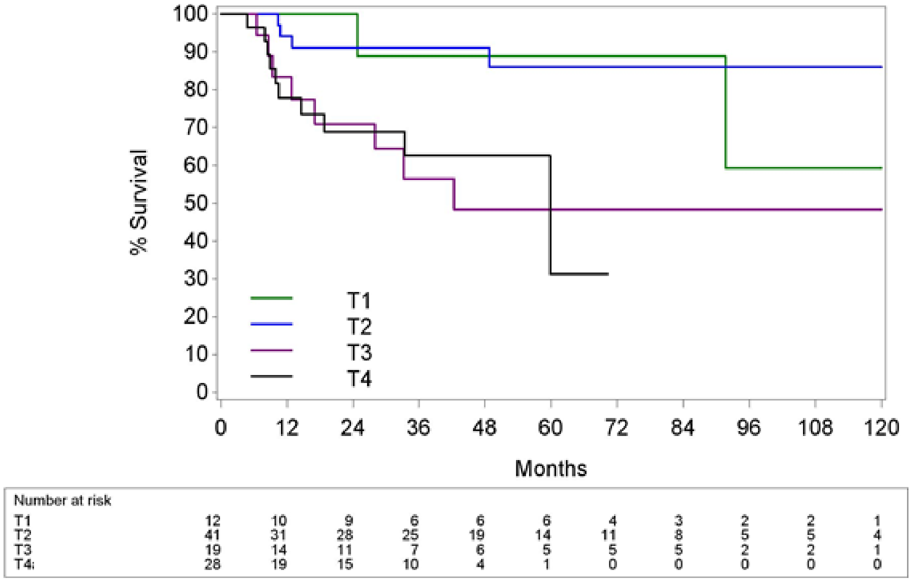

**Comparison**	**p-value**	**Hazard ratio (95% CI)**
**T4 vs T3**	0.942	1.04 (0.40;2.67)
