# Supplementary material for: Patient Selection in Human Papillomavirus Related Oropharyngeal Cancer: The Added Value of Prognostic Models in the New TNM 8th Edition Era
Source: Front Oncol. 2018 Jul 23;8:273. doi: 10.3389/fonc.2018.00273 (PMC6065203; doi:10.3389/fonc.2018.00273)
Supplement: Supplementary file 6 [file Data_Sheet_6.PDF]

Supplementary Material

Title:

Patient Selection in Human Papillomavirus Related Oropharyngeal Cancer: The Added Value of Prognostic Models in the New TNM 8th Edition Era

Running title: Patient-selection in HPV+ Oropharyngeal Cancer

Sarah Deschuymer, Rüveyda Dok, Annouschka Laenen, Esther Hauben, Sandra Nuyts*.

*** Correspondence:** Sandra.nuyts@uzleuven.be

Supplementary Tables and Figures:

S1: Comparison of TNM 7th edition and 8th edition of the T- and N-stages and the number of included patients for each T- and N-stage.
S2: Predictors for overall survival in univariable **(A)** and multivariable **(B)** cox regression analysis.
S3: Patient and tumor characteristics separated by risk group according to the new proposed classification model.
S4: Locoregional control calculated with the cumulative incidence method with death as competing factor for the risk groups defined in figure 10.
S5: Kaplan-Meier curve for overall survival by N-stage **(A)** and T-stage **(B)** according to the TNM 8th edition for HPV positive oropharyngeal squamous cell carcinoma.
S6: Tumor volume of HPV+ OPC according to T-Stage.

S6: Tumor volume (in cc) of HPV+ oropharyngeal squamous cell carcinoma according to T-stage (TNM 8th Edition). Global p-value reporting on differences of tumor volume between the different T-stages was <0.001. There was no significant difference between the tumor volume of T3 versus T4 (p=0.79); HPV: Human papillomavirus; SD: Standard deviation.

	**T1**	**T2**	**T3**	**T4**
N	12	41	19	28
Mean	9.5	14.4	31.5	34.0
SD	8.71	8.85	18.70	21.15
Range	2,2;30.1	1.1;40.4	10.6;76.6	4.9;85.3
